# Mindware: Critical Thinking in Everyday Life

**DOI:** 10.3390/jintelligence12020017

**Published:** 2024-02-02

**Authors:** John Eigenauer

**Affiliations:** Department of Philosophy, Taft College, Taft, CA 93268, USA; jeigenauer@taftcollege.edu

**Keywords:** mindware, critical thinking, artificial intelligence

## Abstract

Humans make many decisions in everyday life, some of which require careful use of evidence. Because emotional and heuristic mental processes dominate human cognition, it is common to suggest that there is little hope that critical thinking tools will be widely used. However, the concept of “mindware” gives hope to the idea that critical thinking skills may be more widely deployed than they currently are. This article reflects on some impediments to critical thinking, assesses some future challenges to critical thinking being more widely used, and suggests that “mindware” modules can be used widely both in and out of educational settings to significantly enhance critical thinking in everyday life.

## 1. Introduction

Humans make thousands of decisions daily, ranging in importance from innocuous to life changing. Many of them involve somewhat automated, habitual, or quotidian matters that require little cognition; however, some of them require deep thought, proper use of evidence, and methods that are generally categorized under the rubric of “critical thinking”. Unfortunately, humans frequently fail to recognize the conditions under which deeper thought, proper evidence, counterfactual thinking, and rules of logic are required for proper decisions. Instead, we over-rely on personal experience, intuition, social cues, and other cognitive shortcuts (Dwyer 2023). Indeed, many of these means are automated. Considerable scholarship acknowledges that automated responses such as these arise outside conscious awareness (Johnson-Laird 2006). For example, Antonio Damasio posits that “we gradually categorize the situations we experience” and that “when a situation that fits the profile of a certain category” is recognized, emotions attached to that category of experiences allow us to “intuit a decision and enact it, speedily and efficiently, without any knowledge of the intermediate steps” (Damasio 2003). And Leonard Mlodinow has documented dozens of experiments in which the unconscious mind strongly influences decision making, ranging from our preference to marry people with our same last name at a much higher rate than would be predicted by probability to the universal ability to recognize human emotion through facial expression (Mlodinow 2012).

The automated nature of human decision making is commonly attributed to the functional efficiency of associative thinking and resource conservation. This fast-processing mode, which has come to be called “System One” processing, allows us to conserve resources through patterned, conditioned replies, often called “heuristics”, that allow us to make decisions, solve problems, and predict outcomes very quickly, without deploying higher-level cognitive systems (Kahneman 2011). Researchers often attribute this “cognitive miserliness” to neurological processes that economize resources (glucose levels or attention span, for example) (Stanovich 2009; Stanovich 2011; Stanovich et al. 2016). In addition, we may take shortcuts from environmental cues, such as cultural or social prompts. Culturally biased responses can manifest as habitual responses to intellectual tasks. And emotions can undermine rational thinking when deeply held personal beliefs, such as political and religious views, are challenged (Wexler 2006; Nisbett 2003; Stanovich 2011). Likewise, humans can bow to social pressure, acting or adopting views in response to perceived or real pressure to conform (Asch 1956; Chen et al. 2022; Schultz 2022).

These automated, System One responses do not involve careful thinking, proper use of evidence, or precise logic and therefore can lead to decisions that are less than optimal or even harmful (Herbranson et al. 2022; Sin et al. 2022). Because these responses are so common, laments about the lack of critical thinking in everyday life are legion. We are accustomed to wondering where commonsense has gone, asking why smart people can do dumb things, and decrying the state of human abilities to make “intelligent” decisions. Those who study this world of thinking know full well that the path to consistent critical thinking is obstructed with seemingly intractable problems like human biases, kneejerk emotional responses, and tribalism (Dwyer 2023). The ubiquity of these responses and the solid science behind them often leads us to conclude that there is no hope that humans will use critical thinking consistently. However, it is possible to reframe this despair into a more sanguine model. In this article, I want to show that one particularly hopeful path to enhancing and broadening critical thinking in everyday life is through the relatively unfamiliar concept of mindware.

## 2. The Concept of Mindware

Keith Stanovich writes that “Mindware is a generic label for the rules, knowledge, procedures, and strategies that a person can retrieve from memory in order to aid decision making and problem solving” (Stanovich 2009). This simple definition, I believe, has the potential to reshape the world of critical thinking instruction and improve critical thinking skills in everyday life. Through this definition, Stanovich suggests that the difficulties of complex decisions and problems can be overcome through acquired modular knowledge and its application using appropriate “rules, knowledge, procedures, and strategies”. In other words, if people could learn these “rules, knowledge, procedures, and strategies”, they would be more successful in recognizing endeavors that require critical thinking and make better decisions in those endeavors. This implies that the seemingly insurmountable roadblocks of social conformity, tribalism, biases, and unthinking emotional replies are merely default techniques for navigating a complex world without the proper thinking tools and that, therefore, with better thinking tools, they could be overcome more consistently.

Reframing critical thinking in this way allows us to see the apparent intractability of heuristic thinking as our way of dealing with complex issues in the absence of more effective tools and not merely as a flaw in its nature—the result of evolutionary development that prioritizes limbic responses. Even though the brain does prioritize limbic responses (Mackeracher 2004), framing the solution to heuristic and emotional responses as “mindware”, that is, as easily learned modules of cognitive strategies, makes possible a graduated approach to critical thinking, emphasizing incremental mastery that begins with easily learnable tools that are applicable to situations that people find useful in the moment. It is unusual that individuals would feel the need to “learn how to think critically”; however, one could see that goals, such as wealth acquisition, weight loss, success at work, or general self-improvement, can all be reached more effectively by using proven rules based on sound evidence and the basic principles of critical thinking.

## 3. Mindware Gaps

Before delving more deeply into mindware, we need to look at one of the most important—and least recognized—principles of human cognition: “unavoidable reality constraints” (Dunning and Kruger 1999). Most people unfamiliar with college mathematics would acknowledge that they do not know how to find the area under a curve using integral calculus, nor would those who never played college football claim that they could compete in the National Football League. That occurs because there is an unavoidable reality constraint to each. The reality of systematic knowledge required to do a calculus problem or the reality of needing world class speed and strength are obvious to everyone. Nonetheless, many people who would acknowledge being unable to do calculus have very strong opinions on how to put the economy right, who would best govern a country, or which belief system guarantees heaven in the afterlife. In a now-famous cartoon from the New Yorker, a man stands up in a plane and declares, “These smug pilots have lost touch with regular passengers like us. Who thinks I should fly the plane?” It is funny because there is an easily recognizable reality constraint to flying an airplane that has escaped the passenger because he has confused expertise with privilege. 

Mindware refers to the presence of “rules, knowledge, procedures, and strategies” that allow for success in many endeavors, such as flying a plane. When we are missing these intellectual skills, we have a “mindware gap”. Mindware gaps are most common in arenas where there are no unavoidable reality constraints. Stanovich introduced the concept of “mindware gaps” to refer to knowledge acquisition and verification procedures that could be used if available but which are not (Stanovich 2009). Notice that Stanovich does not refer to lack of dispositions toward critical thinking or overreliance on default cognitive mechanisms but to procedures that are “not available”. In other words, often what keeps people from using critical thinking—this will sound tautological—is their lack of critical thinking tools. But despite sounding tautological, it is actually a profound shift away from a view that sees critical thinking as a unique skill that few people master toward a view that treats critical thinking modularly. In the same way that we treat learning accounting, mastering a language, or acquiring expertise in nursing, we can treat individual critical thinking skills as discrete and learnable (Maknun 2022; Rarita 2022). If we view critical thinking not as a general inclination, nor as a skill that one possesses or does not, but in a modular way, improvements in critical thinking skills become manageable pedagogically and extendible to people on a broad social scale. Conceptualizing critical thinking modularly would allow people to frame cognitive achievement realistically, in the same way that we acknowledge that there are levels of expertise in wealth management, weight training, or calligraphy. We can begin to see critical thinking as the product of mastering individual skills through concentrated effort rather than a mysterious gift bestowed on the super-rational. 

## 4. “Installing” Mindware: Filling in the Gaps

Let us take the simple idea of probabilistic reasoning. Many thinking errors can be attributed to the failure to think probabilistically: what might be unfortunately labeled the “inability” to think probabilistically (Benjamin et al. 2019). One common error in this arena is the failure to consider base rates (Stanovich 2009). For example, suppose we saw a person in Los Angeles wearing what we considered to be typically French attire—whatever we imagine that to be. Perhaps the person is sporting a beret, wearing a black-and-white striped shirt, and carrying a baguette. What is the likelihood that this person is French? One might use the stereotype to guess that the probability is very high, given the clues, perhaps more than fifty percent. If we guess that that person is French, we would almost certainly be wrong. Why? Because there are 10 million people in Los Angeles and only about 11,000 of them are French: a 99.9% chance of not being French. This tendency to rely on stereotypes and heuristics without reference to statistics is known as “base rate neglect”. In this reasoning error, people over-rely on present experience and individual examples without considering prevalence in a population (Pennycook and Thompson 2022). Most of the mathematical examples used to exemplify this error involve working with probabilities that would intimidate the majority of people. However, the simple rule of remembering to take into account prevalence in a population rather than a very small sample or a stereotype is easy to deploy and very helpful in decision making. For example, very few people who want to be professional actors, social media influencers, or YouTube stars recognize that only an extremely small percentage of people who hope to succeed in those fields attain even a small degree of success. Teaching probabilities and base rates may seem like a daunting task. However, there are games that teach its fundamental principles, such as BeatTheOdds, Probability Fair, TinyTap Probability, and The Vile Vendor. Each game enhances probabilistic thinking mindware.

Humans are also generally ill-disposed to seek answers to questions once an adequate answer has been found. This tendency has been described in various ways in the literature from cognitive science. For example, in radiology, “satisfaction of search” occurs when the physician “fails to continue to search for subsequent abnormalities after identifying an initial one” (Knipe et al. 2021; Ashman et al. 2000). Similar tendencies have been noted in airport security workers, leading to the inference that this may be a general cognitive tendency. As Mirtoff, Biggs, and Cain write, “Over 50 years of research has shown that when searchers successfully find a visual target, they become less likely to find another target in the same” (Mitroff et al. 2015). Clearly, this research has implications not only for radiology and security checks but for crime scene investigation, loss prevention, logistics, drug interventions, psychological diagnoses, psychological treatments, instructional methodologies, business growth, and many other fields. The particular mindware would be the simple rule that when one finds an answer, a malfunction, a sought object, or a piece of evidence, one should continue searching to find additional answers, problems, objects, or evidence. “Installing” the mindware in the minds of students, employees, or citizens would be a simple matter of raising awareness regarding the cognitive tendency to cease searching and the necessity to continue the search even after an initial target is acquired. 

Closely related to this tendency of satisficing (Caplin et al. 2011) is the idea of a “makes-sense epistemology”. The term, coined by David Perkins, describes the tendency to accept as true any explanation that has an adequate feel of plausibility (Perkins et al. 1991). Our brains have not evolved to be constant truth-seeking machines; instead, they rely on good-enough explanations—approximations that allow us to move forward with decisions based on incomplete knowledge. Most of the time, such decisions are not dangerous and can, indeed, be trusted to lead us down a path that will provide some degree of happiness, success, pleasure, or correctness. However, in more-important matters, such as decisions that inform public policy, responses that rely on personal experience, associative thinking, and heuristics are nearly always inadequate. However, a simple piece of mindware allows us to circumvent the mistakes that follow from satisfactory explanations generated by System One. This is the habit of considering alternative explanations. For example, during the height of the COVID-19 pandemic in the United States, hospitals filled up at highly variable rates. It was important to know why this was happening because the differences might have supplied clues as to how to improve hospitalization rates or allow local areas to respond more effectively. Rather than reducing the issue to a bipartisan political problem (as it was sometimes presented), it was important to recognize that numerous factors might have influenced occupation rates. The following are only some: local vaccination rates; the number of available ICU beds; the general health of the local population; underlying health conditions in the population; prevalence of smoking; age of the local population; distance to hospitals; and the concurrence of other disasters, such as weather events. This simple skill could easily become habituated among students, for example, through simple and consistent assignments in considering alternative explanations. Asking students to formulate a variety of hypotheses about what might explain recidivism, wealth acquisition, or athletic prowess would cultivate habits of mind that could be consistently deployed in a variety of fields and significantly enhance critical thinking (Alsaleh 2020). One can even imagine a game show called “Alternative Explanations” along the lines of the format of the famous “Family Feud”.

## 5. Taking Mindware Public

However, only a small percentage of the population has access to direct instruction in critical thinking. Therefore, the question remains about how a society might leverage the concept of mindware to enhance critical thinking in broader sectors of the population. Mindware’s modularity helps solve this problem. Moreover, our knowledge of how difficult it is to induce belief change can be used to an advantage. For example, learning that one needs to learn to think critically can be threatening, especially since such comments are commonly made in reaction to the perception that one is not doing so. This issue is even more intractable in highly personalized realms, such as politics and religious discourse. Other areas are more approachable. For example, many people are interested in retirement planning, often making significant errors on the road to retirement or over-relying on the wrong professionals. However, retirement planning is a piece of mindware whose principles are readily “installed”: begin saving money early, use compound interest, avoid debt, and scrutinize luxury purchases. At the risk of promoting a particular brand of retirement planning, Dave Ramsey’s work promotes these basic steps toward wealth accumulation (Ramsey 2013). They are not, however, the unique ideas of an investment guru—they are fundamental economic principles that Ramsey has successfully condensed into a format that anyone can follow. He popularized a piece of mindware. In essence, he reduced a seemingly difficult problem—retirement planning—to a set of “rules, knowledge, procedures, and strategies” that could be easily learned, remembered, and practiced.

In order for people to recognize that this is a practice of critical thinking in everyday life, methods such as Ramsey’s need to be labeled as such. If Mr. Ramsey were to refer to his program as a piece of “mindware” that promoted wealth acquisition and critical thinking (defined here as following known rules for a positive benefit while eliminating errors caused by emotion and reliance on heuristics), the practical and beneficial aspects of following logical rules to reach a desired end would be more obvious to the population at large. 

## 6. Contaminated Mindware

Despite the relative ease of learning/installing mindware, it can be offset by contaminated mindware. Just as mindware is a set of knowledge, skills, and procedures that lead to more rational, beneficial, or epistemically sound decisions and conclusions, contaminated mindware leads to the opposite. While there are many varieties of contaminated mindware, such as superstition and anti-scientific beliefs, Stanovich cites “mindware that contains evaluation-disabling procedures” as particularly pernicious. These include “the promise of punishment if the mindware is questioned; the promise of rewards for unquestioning faith in the mindware; or the thwarting of evaluation attempts by rendering the mindware unfalsifiable” (Stanovich 2011). Any faulty thought processes may be considered to be contaminated mindware as well: racism, xenophobia, radical skepticism, human sacrifice, or even public policy in a democracy that fails to consider data, demographics, or current scientific knowledge. These “evaluation-disabling procedures” map to activities that disallow critical thinking. Indeed, anything that proscribes evidence, questioning, and logic in the name of the sacredness of the ideology falls into the category of “contaminated mindware”.

The fact that mindware can not only be contaminated but spread easily once contaminated presents one of the greatest challenges to the dissemination of critical thinking skills to society at large. The intractability of utter conviction has been recognized for thousands of years (Beck 2017), and the absolute fact of belief enhancement in the face of contrary evidence has been firmly established by cognitive scientists (Susmann and Wegener 2023), social psychologists (Gold and Gold 2014), and philosophers. Writers have puzzled over how to convince others of their obviously erroneous ideas, sometimes despairing of the hopelessness of logical argumentation and the inefficacy of evidence to change minds (Staats et al. 2017). The psychologist Lou Cozolino explains this tendency humorously by pointing out the difference between rats and humans. Rats, he says, will find cheese in a maze and return to the spot where they found it; when it is no longer there, they look elsewhere. “Humans, on the other hand”, he says, will return to the original spot “forever because they come to believe that’s where the cheese should be. Within a few generations, humans will develop rituals, philosophies, and religions focused on” the cheese’s original place, and “invent gods to rule over it” (Cozolino and Davis 2017).

## 7. Critical Thinking, Mindware, and Artificial Intelligence

Possibly the greatest threat to critical thinking in everyday life, however, lies in the near future: artificial intelligence—especially knowledge generators such as ChatGPT. Starting in the 1980s, authors such as Neil Postman began raising the cry against the potential negative effects of automated technologies that infringed upon thinking processes. Postman warned that beginning in the early twentieth century, “workers were relieved of any responsibility to think at all. The system would do their thinking for them”, with the disastrous consequence of technology moving into the realm of the “moral, social, and political”. This led to the “idea that society is best served when human beings are placed at the disposal of their techniques and technology” (Postman 1993). With the rise of the Internet, others began recognizing that many human thought processes could be delegated to software and intelligent machines, just as manual labor had been automated through machinery and robotics. Nicholas Carr, among others, warned of the deleterious effects of human overreliance on computational devices (Carr 2010, 2014; Lanier 2013). Studies such as “Google Effects on Memory” (Sparrow et al. 2011) and Dahmani and Bohbot’s (2020) study revealing the negative effects of habitual GPS use demonstrate the cognitive and neurological tradeoff between the efficiency of increased computational capacity and the slower cultivation of organic brain power. But artificial intelligence presents to us the possibility of not needing to think, weigh evidence, make decisions, and perform many cognitive tasks that make us human. It presents the very real possibility of providing an automated substitute for critical thinking in everyday life in the same way that GPS provides an automated substitute for navigating, search engines for laborious research, and, at times, social media for real-life interactions.

An important artificial intelligence manifesto is Marko Rodriguez’ 2009 article (Rodriguez and Watkins 2009) titled “Faith in the Algorithm, Part 2: Computational Eudaemonics”. In it, he argues that as a matter of social responsibility and in order to maximize happiness for humanity, we should turn our decision making over to machines—to the algorithm. He writes that once a certain computational capacity is reached and combined with massive data, we can have

“a society of individuals where the vocation one takes, the person one dates, the books one reads, the restaurants one frequents, and so on are chosen not through the advice of one’s family, friends, and community, but through a deep computational understanding of what is required for that individual to live an optimal life…. In other words, the individual would choose options that they do not perceive as necessary. Without the perception of need, the individual would take on faith that the algorithm knows what is best for them in a resource complex world. Thus, the perfect life is not an aspiration, but a well-computed path”.

Written fourteen years before the release of ChatGPT, the article nicely summarizes the true potential of AI in the realm of human life, thinking, and decision making. Artificial intelligence (“algorithms” in Rodriguez’ parlance) is the computational mindware whose function it is to bypass the inefficiency of human cognition. By using AI, humans subcontract the knowledge acquisition and verification procedures of critical thinking, creativity, and decision making (Marszalek-Kotzur 2022). Reliance on AI for these processes may circumvent the need for critical thinking in the same way that faith in any given ideology circumvents the need for analysis, evaluation, and evidence. In other words, AI has the potential to create the ultimate mindware gap by eliminating the need for mindware. The knowledge, skills, procedures, and protocols that make up human mindware could be transferred to the black box of artificial intelligence. In the same way that people do not remember facts so much as remember where they can find the fact when Internet access is available to them, people may not remember and utilize the mindware necessary for proper thinking and decision making so much as remember where the mindware resides: in the AI. 

Many comparisons are being drawn between technologies that replaced human labor and AI. Those who decry the intrusion of AI into human thought and creativity are sometimes labeled “Luddites”. Long before, Plato wanted to ban writing because it might supplant human memory; the case of AI could be qualitatively different. We now have the possibility to use AI to write papers (Terry 2023), thereby eliminating the need for research and effortful thinking. AI is already used as a “truth generator” despite its embarrassing failures (Neumeister 2023), which could be seen as the technological equivalent of an ideology generator. The mindware at the heart of critical thinking—questioning the truth of claims and evaluating the viability, motivation, and evidence behind ideologies—could be rendered inoperable by trusting AI to generate answers. Like ideologies, AI qualifies as having “evaluation-disabling procedures” implied in its apparent infallibility. 

## 8. Ideologies and Artificial Intelligence

Since AI may infringe upon independent human thinking and circumvent gathering and evaluating evidence, it appears to function as do ideologies. Leor Zmigrod has suggested that ideological thinking is “a style of thinking that is rigid in its adherence to a doctrine and resistance to evidence-based belief-updating” (Zmigrod 2022). Seeking to understand the nature of “ideological cognition” rather than the supposedly convincing nature of “the content of ideological beliefs”, Zmigrod concludes that ideological thinking is “a meaningful psychological phenomenon”. While there are no studies about the psychology of reliance on AI for answers, other studies on our reliance on search engines, GPS, and other technologies, combined with our knowledge of default modes of thought (System One thinking, usage of heuristics, cognitive miserliness), offer viable reasons to believe that AI will quickly take its place alongside these other technologies in diminishing the perceived need for effortful thought. As such, AI use—especially as a knowledge generator—could soon be considered “a meaningful psychological phenomenon”. In other words, they will be using “evaluation-disabling procedures” as easily and frequently as they use search engines and GPS. This may be best understood by recognizing that GPS is a “navigation-disabling procedure” to the degree that it is used as a “navigation-facilitation” tool. In an age of increased nationalism (Brown 2022), ideological influence in healthcare decisions (Ruisch et al. 2021), ideological divides surrounding climate change (Ballew et al. 2020), moral and ethical issues (Voelkel and Brandt 2019; Waytz et al. 2019), and, of course, religion (Perry 2022), if humans turn to knowledge generators such as ChatGPT to help formulate beliefs in unfamiliar areas, regaining control over our own ideas is all the more necessary. 

## 9. Mindware to Promote System Two Thinking

One of the greatest advances in cognitive science in the twenty-first century has been the recognition that the human brain’s default mode uses heuristics, social cues, cultural norms, and resource conservation when positing answers, reaching conclusions, and making decisions. This research has enabled increasingly precise insights into tribalistic thinking, myside bias, cultural divisiveness, and a broad array of thinking errors. Conceptualizing critical thinking in everyday life as consisting in part as “mindware modules” holds great promise to help people circumvent the problems created by reliance System One thinking when more effortful thinking is required. Once people learn the concept of mindware and the accompanying concepts of mindware gaps and contaminated mindware, awareness can motivate people to use the rules, knowledge, procedures, and strategies of rationality and succeed in implementing critical thinking as mindware. Once the vocabulary is adopted, school courses could be developed titled “mindware for retirement”, “mindware for public policy”, “mindware for crime scene investigation”, “mindware for healthcare professionals”, and the like. Thus, the terms “mindware for retirement”, “mindware for public policy”, and the like could be recognized as the “rules and strategies” that anyone can learn to increase the likelihood of success in these fields. In the same way that people can learn to use accounting software to help with a small business, they could learn the rules and strategies for college success, retirement planning, or environmental responsibility. Thus, the vocabulary of mindware could be spread outside academia as well.

## 10. Conclusions

The concept of mindware as learnable “rules, knowledge, procedures, and strategies” has the potential to enhance awareness about the need for critical thinking and to provide individuals with easily learnable tools to make better decisions, be better thinkers, and use evidence more consistently and virtuously. Mindware’s individual modules (retirement planning, weight loss, relationship enhancement, college success) then become manageable units of decision enhancement that lead to better critical thinking abilities. As AI makes inroads into everyday life, offering people solutions to decisions without the customary cognitive effort, the concept of mindware and the implementation of mindware modules may help us maintain and enhance individual CT abilities.

## Data Availability

Not applicable.
